# The Synthesis of Organic Oils Blended Magnetorheological Fluids with the Field-Dependent Material Characterization

**DOI:** 10.3390/ijms20225766

**Published:** 2019-11-16

**Authors:** Rakesh Jinaga, T. Jagadeesha, Shreedhar Kolekar, Seung-Bok Choi

**Affiliations:** 1 Department of Mechanical Engineering, National Institute of Technology, Calicut 673 601, India; jagdishsg@nitc.ac.in; 2 Department of Mechanical Engineering, Satara College of Engineering & Management Limb, Satara 415 015, India; shreedharkolekar@gmail.com; 3 School of Mechanical Engineering, Inha University, Incheon 22212, Korea

**Keywords:** magnetorheological (MR) fluid, organic oil, carrier liquid, eco-friendly MR fluid, guar gum, rheological property, sedimentation stability

## Abstract

Automation is one of the trending terminologies in the field of engineering to achieve various sensors and actuators such as the hydraulic system. Smart fluid is also one of the hot topics for researchers to develop a type of actuator in many control systems since the fluid’s rheological characteristics can be controlled or tuned by the intensity of the external stimuli. In this work, a new smart fluid of magnetorheological (MR) fluid is proposed and its field-dependent rheological characteristics are experimentally identified. An MR fluid using the carrier fluid as the blend of three different fluids, namely silicon oil, honey, and organic oil is prepared. In addition, two types of natural oils are used, sunflower oil and cottonseed oil. The samples are prepared using the blend as the carrier fluid, electrolytic iron powder coated with guar gum as the dispersed phase, and oleic acid as an additive. The quantity of oleic acid is optimized for 30% by weight of electrolytic iron powder. Two samples based on sunflower and cottonseed oil are synthesized and characterized for shear viscosity and shear stress with respect to shear rate subjected to a variable magnetic field. The blend-based MR fluid shows about 10% improvement over the sedimentation rate of silicon oil-based MR fluid as compared to that to conventional MR fluid. The cottonseed oil blend-based MR fluid performs better than sunflower-based fluid in terms of the viscosity and structure.

## 1. Introduction

Magnetorheological (MR) fluid is one of the kinds of smart fluids where the behavior of fluid can be shifted from Newtonian fluid to a semi-solid material (Bingham fluids) by being subjected to a magnetic field. The rheological properties of MR fluids are the function of an applied variable magnetic field; hence, to obtain precise control over rheological properties a variable magnetic field is desirable. Additionally, the yield stress in MR fluid which is directly proportional to the viscosity and intern to the applied variable magnetic field, can be formulated as [1].

(1) $$\tau =\tau_{y\left(H\right)}+\mu_{p}\dot{\gamma }$$

where, $\;\tau_{y\left(H\right)}$ is the yield stress as a result of the applied variable magnetic field H, $\mu_{p}$ is the plastic viscosity constant and $\dot{\gamma }$ is the the shear strain rate. The various physical properties of the MR fluid are influenced mainly by the applied variable magnetic field.

Synthesis of fluid dates back to 1949 by Jacob Rabinow, further the development of fluid was carried out in various aspects and stages. Sarkar and Hirani [1] carried out a study to enhance the dispersibility of the carbonyl iron (CI) particles suspended in the carrier fluid in order to bring down the sedimentation rate of magnetic particles in fluid. Fang et al. [2] presented a single-walled carbon nanotube in a CI-based magnetorheological fluid to enhance sedimentation time. Shetty and Prasad [3] manufactured magnetorheological fluid using vegetable oils (nonedible) as the carrier fluid and concluded that the yield stress obtained drops drastically reaching 25 kPa. Hirani [1] deliberated the process of the synthesis of MR fluid, considering its braking point. Cho et al. [4] encapsulated CI particles using poly-methyl methacrylate. Jiang et al. [5] supplemented wire-like iron nano-structures to the conventional MR fluid with CI particles leading to a dimorphic MR fluid. Fang et al. [6] synthesized the guar gum-based MR fluid by the process of ball-milling guar gum powder along with carbonyl iron powder and silicone oil. The results showed that the presence of guar gum enhances the thixotropy and sedimentation stability effectively. Sedlacik et al. [7] employed the method of coating a thin layer of 3APTS with a grafting density of 50 groups/nm^2^, and identified improved compatibility of non-polar silicone oil with coated particles. Kumar [8] studied the characterization of honey oil-based MR fluid for the brake applications, whereas the degradation and stability of the fluid were not critically identified. It showed that the use of silicon oil and honey together could promise better stability and resistance to degradation. The presently available commercial MR fluids limit their application in common usage because of their high pricing and the environmental hazards caused by the ingredients. The environmentally friendly and cheaper MR fluid shows poor performance lacking the fundamental physical properties such as off-state viscosity [8].

In this study, the basic and critical constituent of MR fluid, that is the, carrier fluid is the center of importance which is the blend of organic oils. For the study, two organic oils are considered, namely, sunflower oil and cottonseed oil. The blends are prepared using fluid silicon oil (25%), honey (25%), and organic oil (50%) weight ratio. An electrolytic iron powder (EC10TR) is employed as magnetizable particles, these particles are coated with guar gum to enhance dispersibility and finally the oleic acid which forms a bond between the guar gum film and a carrier fluid is added. In this study, the carrier fluid used employs organic fluid (75% by weight), which ensures the eco-friendly nature of the MR fluid. The obtained MR fluid makes it a right candidate for various MR applications such as brake, clutches, damper, etc.

## 2. Synthesis of MR Fluid

The manufacturing process of MR fluids follows an exclusive process involving the coating of magnetic particles with guar gum and also obtaining a carrier fluid by blending various fluids to enhance the physical properties of MR fluid [6,9].

### 2.1. Required Properties

The physical and rheological characteristics of a magnetorheological fluid are the combined effort of each constituent put together in the process of synthesis. The optimization of these parameters and constituents for various applications is one of the biggest challenging issues for research. In this paper, an effort was made to optimize the additive in synthesis process. There are various parameters and factors which influence the overall performance of MR fluid, out of these the critical aspects are discussed below.

#### 2.1.1. Lower Viscosity at Off-State

In order to obtain a high performing MR fluid, a researcher has to consider off-state viscosity of MR fluid as a critical parameter. The field-independent or off-state viscosity (η) is one of the critical characteristics of MR fluid, as the whole system is expected to operate under this viscosity of fluid. This property of fluid is largely influenced by two parameters, one being the volume fraction of magnetizable particles, and the other is the intrinsic viscosity of the carrier fluid or base fluid [10]. Of the conventional MR fluids which find a wide range of application, Lord MRF 132DG possesses an off-state viscosity of 0.13 Pa.s, at a slope of 800–1200 sec^−1^ maintained at 40 °C [11]. The field-independent viscosity of MR fluid is directly proportional to its volume fraction of the magnetic particles dispersed in a fluid.

#### 2.1.2. Higher Yield Stress at On-State

Under the magnetic field, the yield stress of the MR fluids is boosted substantially due to dipole–dipole formations among particles dispersed in a fluid. The yield stress is a critical parameter in the case of MR application as the value of yield stress under the magnetic field is directly proportional to that of resisting torque generated by the system. The yield stress of MR fluid increases with an increase in the volume fraction of particles, and on the other hand, increases off-state viscosity. Hence, an optimal volume fraction of dispersed magnetic particles is critical. In addition, the material of the magnetic particles plays an essential role in attaining maximum yield stress, as it is proportional to the square of saturation magnetization of magnetic particles [12,13]. The literature shows the yield stress value varies with respect to applied magnetic field and shows a nonlinear increment with the increasing volume fraction of particles [5,14,15]. Lord MRF 132DG possesses a yield strength of 48 KPa subjected to 288 kA/m [11]. In order to obtain better performance of the magnetorheological system, high yield stress MR fluid was preferred.

#### 2.1.3. Presence of External Magnetic Field

The important characteristics of magnetorheological fluids, which make it so versatile and flexible to fit in and to resolve most of engineering applications problems, is the ability to vary the viscosity as a function of applied magnetic fields. The strength of the magnetic field determines the nature and behavior of MR fluid, varying from Newtonian to non-Newtonian fluids such as the Bingham fluid. Numerous studies have been carried out to bring out the field-dependent behavior of magnetorheological fluids. More specifically, it was shown that the viscosity of 8.7 Pa.s was measured at 0.04 T and 45 Pa.s at 0.1 T, respectively [14]. On the other hand, it has been proven that MR fluids, which are currently commercial, can be operated ranging from −20 °C to 160 °C [11]. This controllable characteristic behavior in a wide temperature range is the salient factor for applications of MR fluids to various control systems such as the automotive damper.

#### 2.1.4. Reduced in Use Thickening

One of the disadvantages of MR fluids applications is its thickening when it is subjected to higher shear rate and stress over a long time. The phenomenon is addressed as In Use Thickening (IUT) and is one of the challenging aspects for automobile industries [12,13]. As a result of IUT, the off-state viscosity of fluid increases leading to drag and increased power loss under off-state conditions.

#### 2.1.5. Wider Temperature Range

As the application of MR fluid-based systems in the automobile sector is of importance and the operating temperature of these automobiles can range from sub-zero to prolonged operating time resulting from temperature rise. Commercially available MR fluid is reported to withstand temperature of 80 °C.

### 2.2. Organic Oil Blends

The carrier fluid plays a critical role in deciding the characteristics of the MR fluid as it forms the major essentials of fluid (50%–80% by volume) [3]. Commercially used carrier fluids are silicon, mineral, and synthetic oils. In this study, the carrier fluid is prepared by blending three fluids namely silicon oil 25% by weight, honey 25% by weight, and sunflower/cottonseed oil (organic oil) 50% by weight. The presence of silicon oil and honey improves the stabilization of organic oil over all contributing to achieve the required off-state viscosity of MR fluid. The large part of organic oil contributes to the eco-friendly nature of the MR fluid and provides compatibility with guar gum which is used as coating for electrolytic iron powder. The previous study [8], where a comparative study of MR fluid with carrier fluid as honey-based MR fluid and silicon oil-based MR fluid over an MR brake system is undertaken, concludes that the honey-based MR fluid provides better braking torque. The constituent of organic fluid which adds 75% by weight makes the fluid environmentally friendly. The physical properties of constituents of the prepared blend are listed in Table 1; Table 2, respectively. The dynamic viscosity was determined by using an Anton Paar Rheometer, while the density using conventional method of weight by volume ratio, whereas the flash and fire point was measured using the Pensky–Martens flash point apparatus. It is remarked here that the experimental work conducted in this study was undertaken at a room temperature of 25 °C. The physical properties of carrier liquids such as sunflower oil and honey are provided in Table 1 and Table 2. As seen from Table 1, the flash and fire points of the honey only is very low compared with the others. Therefore, in this work the blended carrier fluids given in Table 2 are used to characterize the properties of MR fluids.

### 2.3. Magnetic Particles

Commonly the particle size of magnetizable particles dispersed in MR fluid varies from 1 to 10 µm [2,16]. The sedimentation rate is a function of particle size, that is, with increase in particle size the sedimentation rate increases, hence to obtain optimum effect EC10TR electrolytic iron powder from Industrial metal powder (India) Pvt. Ltd. A particle size distribution ranging from 1 to 10 µm with an irregular shape and size resembling rod-like structures was selected for the study. The magnetic particles used for the synthesis of samples were taken as 30% by weight. The off-state viscosity is directly proportional to the weight fraction of magnetic particles. On the other hand, the increased quantity of particles enhances yield stress of fluid which is very much desirable in the MR application. The samples are prepared with various combinations of electrolytic iron powder, the off-state viscosity of the samples prepared with sunflower oil as organic oil and 30% (weight ratio) of electrolytic iron powder was obtained within 0.5 Pa.s. Whereas cottonseed is comparatively viscous, sunflower oil shows increased off-state viscosity as compared to samples prepared from sunflower oil as biodegradable oil with the same composition; hence having better stability towards sedimentation.

### 2.4. Additives

The stabilizers are employed in order to keep the magnetizable particles suspended in the carrier fluid, whereas surfactants serve in enhancing polarization by adhering to the surface of magnetic particles under the applied magnetic field. In this study, guar gum is used as a coating to magnetic particles that provides antifriction behavior to the application system. Hence, this enhances sedimentation stability [6,9]. Furthermore, oleic acid is employed to serve as a stabilizer in order to have better rheological characteristics and sedimentation stability [17,18,19]. For this study, guar gum is employed for coating the electrolytic iron (EI) particles and oleic acid as a stabilizer.

### 2.5. Manufacturing Process

The preparation of MR fluid starts with the preparation of carrier fluid, that is, the organic oil blend and the coating of magnetic particles using guar gum. Two types of organic oil blends are prepared (1) silicon oil (25%) + honey (25%) + sunflower oil (50%), called sunflower-based blend, and (2) silicon oil (25%) + honey (25%) + cottonseed oil (50%), called cottonseed-based blend. The electrolytic iron powder particles are used as magnetic particles in the study, the coating of electrolytic iron powder is done using guar gum, the irregular shape and surface texture of EI particles promotes proper surface interaction for coating.

**Figure 1:**
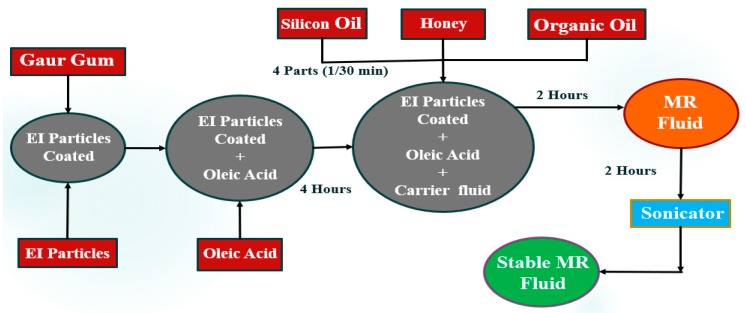
The manufacturing process of magnetorheological (MR) fluids featuring various liquid oils.

The coated magnetic particles are mixed with oleic acid to obtain proper bonding between oleic acid and the guar gum present on the surface of EI particles. The weight proportion of EI powder in prepared samples is kept constant to 30-weight percentage, and the proportion of oleic acid is varied to investigate the best suitable amount of oleic acid for EI composition. After the mixture is ready, it is supplemented with the prepared organic oil-based blend carrier fluid in parts of four for every half hour, which helps the thorough mixing and dispersion of the particles in carrier fluid, followed by two hours of stirring. The prepared MR fluid is subjected to sonication for two hours to confirm uniform dispersion of particles in the fluid to obtain a stable MR fluid. The pictorial representation of the whole process is shown in Figure 1. The obtained MR fluid with 30-weight percentage coated EI powder, blended carrier fluid, and optimized oleic acid are subjected to different magnetic field strengths to investigate the performance.

## 3. Material Characterization

### 3.1. Analysis of SEM Images

**Figure 2:**
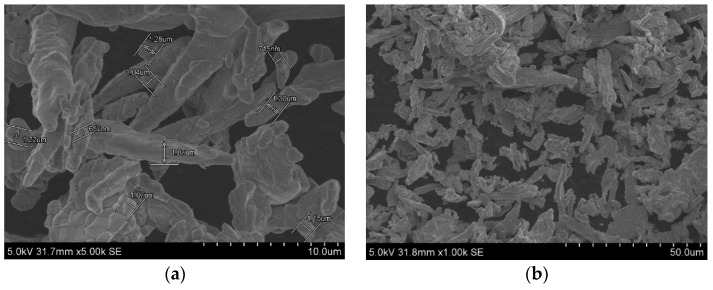
SEM images of electrolytic iron powder with a rod-like structure with irregular shape and surface; (**a**) electrolytic iron (EI) particles shape and size, (**b**) particles coated with guar gum.

The scanning electron microscope (SEM) is used to study the morphological characteristics of the sample, and hence the quality of particles is tested. The details on the structure of particles and the size distribution carried out using SEM is presented in Figure 2. The structure of the particles is rod-like, 1–2 µm in diameter, and has an average size ranging from 1 to 10 µm. The irregular shape and size of the particles helps in superficial interactions with guar gum and makes it a better dispersant. The yield strength value of the particles is increased because of the asymmetrical rod-like structures of the particles creating a dipole–dipole interaction with the surrounding particles.

### 3.2. Shear Stress versus Shear Rate

**Figure 3:**
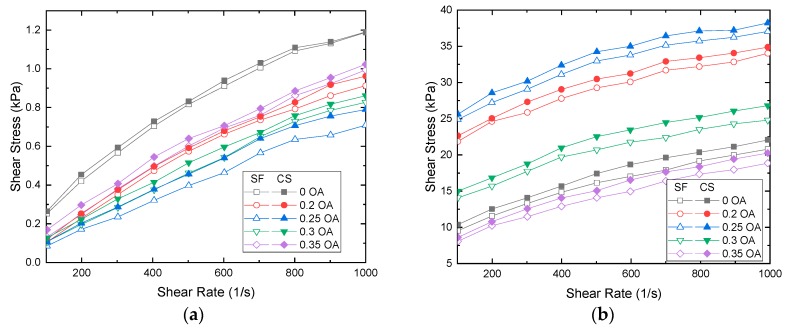

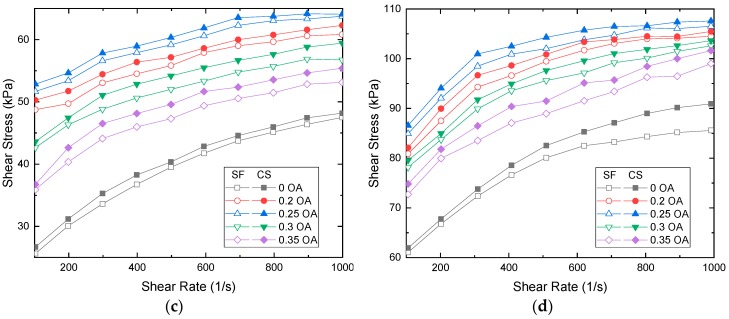
Shear stress vs. shear rate for MR fluids with a variable composition of oleic acid subjected to different magnetic fields. (**a**) 0 kA/m, (**b**) 45 kA/m, (**c**) 81 kA/m and (**d**) 136 kA/m.

The study was carried out for two batches of MR fluid samples and in each sample series, five different samples for each batch were synthesized by varying the weights of the oleic acid (OA) ranging from zero to 0.35% (wt). The optimal weight percentage of OA in the MR fluid composition is identified by plotting graphs between the shear rate and shear stress of the samples, and is presented in Figure 3. The characterization is carried out using an Anton Paar-make Rheometer MCR 102 with a magnetic chamber attachment MRD 170/IT to generate a magnetic field. To optimize the weight percentage of (OA) in MR fluids, samples with a series of weights percentage like 0, 0.2, 0.25, 0.3 and 0.35 are prepared for both Sample 1 and 2 with a total of 10 samples.

The results for shear rate varied from 100 to 1000 s^−1^ as a function of shear stress are plotted in Figure 3. From Figure 3a it is proved that the addition of oleic acid reduces the value of shear stress when the magnetic field is 0 kA/m. The presence of OA in the sample leads to a reduction in shear stress value as compared to a sample without OA. This occurs because of free suspended magnetic particles in the fluid as the sample comes under the influence of magnetic field where the particles align. The samples are exposed to different magnetic field strengths of values 45 kA/m, 81 kA/m, and 136 kA/m, and the results are presented in Figure 3b,d. As the sample with 0.25% of OA performs better under all applied magnetic fields and other proportions of OA for both sunflower and cottonseed oil-based samples. Hence, the samples with 0.25% of OA stand out as the best samples. Therefore, it can be concluded that 0.25% of weight with OA offers lower off-state viscosity and high on-state viscosity.

**Figure 4:**
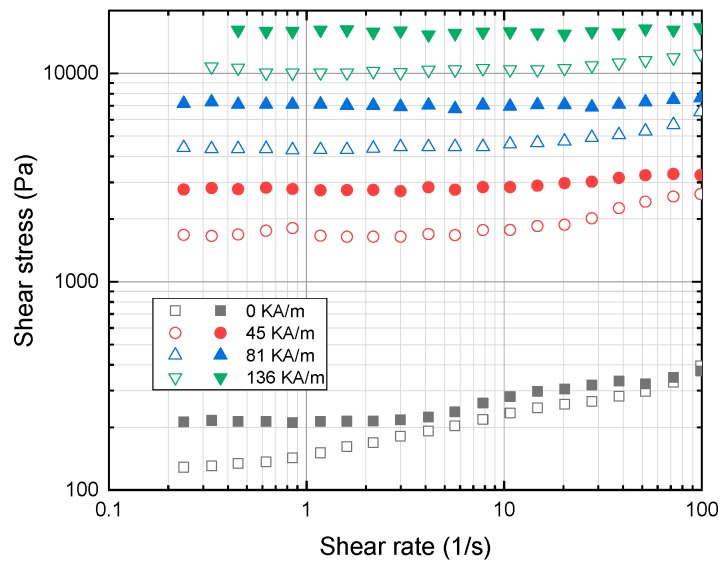
Shear stress as a function of shear rate for sunflower (open symbol) and cottonseed oil (closed symbol) blend-based MR fluid subjected to variable magnetic field i.e., 0, 45, 81 and 136 kA/m.

**Figure 5:**
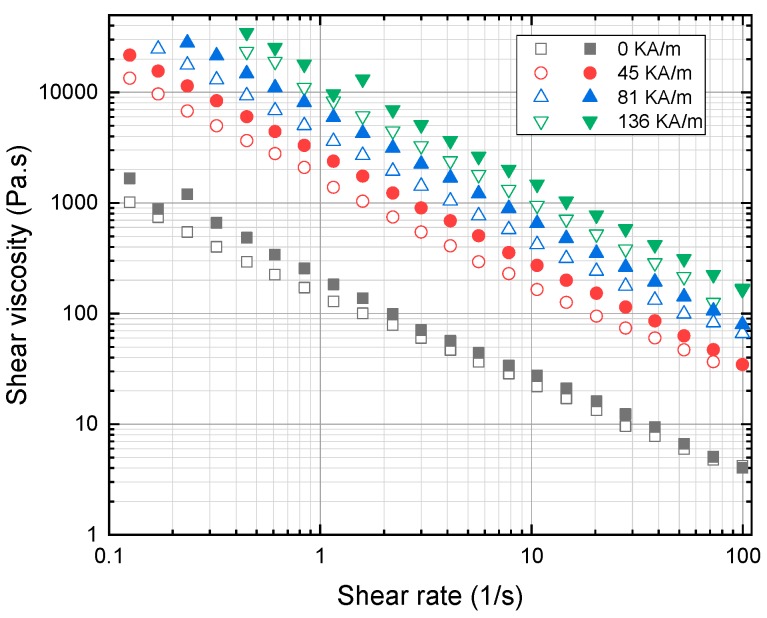
Shear viscosity as a function of shear rate for sunflower (open symbol) and cottonseed oil (closed symbol) blend-based MR fluid subjected to variable magnetic field i.e., 0, 45, 81 and 136 kA/m.

**Figure 6:**
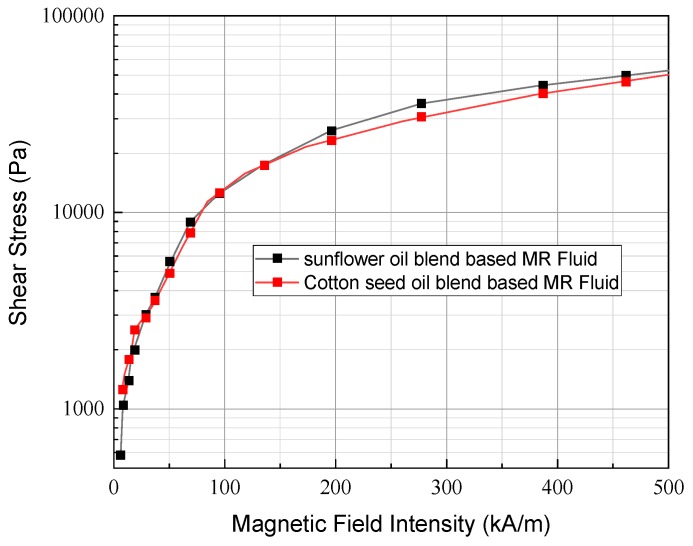
Shear stress as a function of a variable magnetic field for the organic oil blend-based MR fluids.

With the optimized quantity of additive OA, the final samples were prepared with blends of cottonseed oil and sunflower oil. It is noted that the rheological characterization of the MR fluid for the samples is carried out under a static magnetic field and constant static shear stress. The plot in Figure 4 shows shear stress as a function of shear rate, and Figure 5 shows shear viscosity as a function of shear rate at four different values of the magnetic field: 0, 45, 81 and 136 kA/m. The yield stress value is obtained by extrapolating shear stress values to zero shear rate. It is identified from the result that the yield stress at 136 kA/m for cottonseed- and sunflower-based fluid is 20.13 and 17.73 kPa, respectively. In addition, it is evident from the plot that in order to increase the shear stress level along the shear rate region. The strength of the magnetic field must be increased. Both the samples exhibit Bingham plastic behavior with a non-vanishing stress value under the applied magnetic field which shown in Figure 6. This shows that the MR fluid sample forms a stable chain-like structure created by the dipole interaction of particles for the shear rate ranging from 100 to 1000 s^−1^. The sample exhibits a non-Newtonian behavior, even under off-state conditions which is due to the high concentration of magnetic particles in the samples. The off-state viscosity of cottonseed oil-based MR fluid is slightly higher than sunflower oil-based fluid because of its inherent high viscosity.

### 3.3. Field-Dependent Loss and Storage Modulus

**Figure 7:**
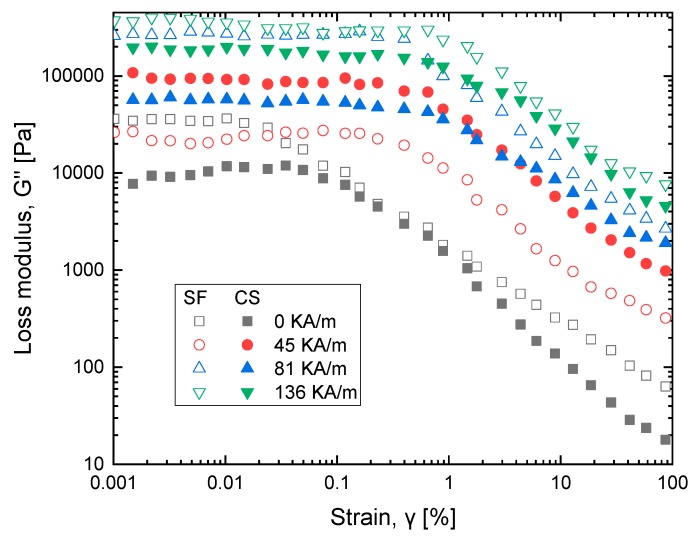
Loss modulus as a function of strain at variable magnetic field strength for sunflower (SF) and cottonseed (CS) blend-based MR fluid.

**Figure 8:**
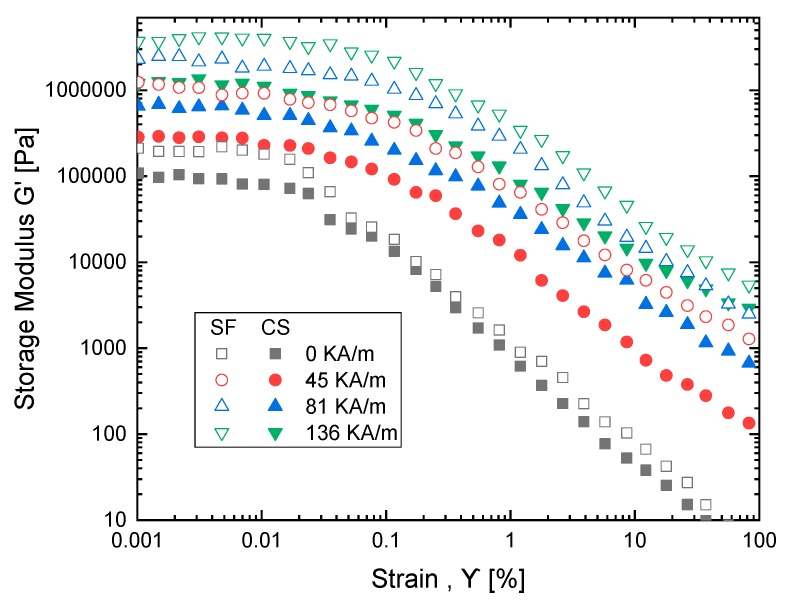
Storage modulus as a function of strain at variable magnetic field strength for sunflower (SF) and cottonseed (CS) blend-based MR fluid.

**Figure 9:**
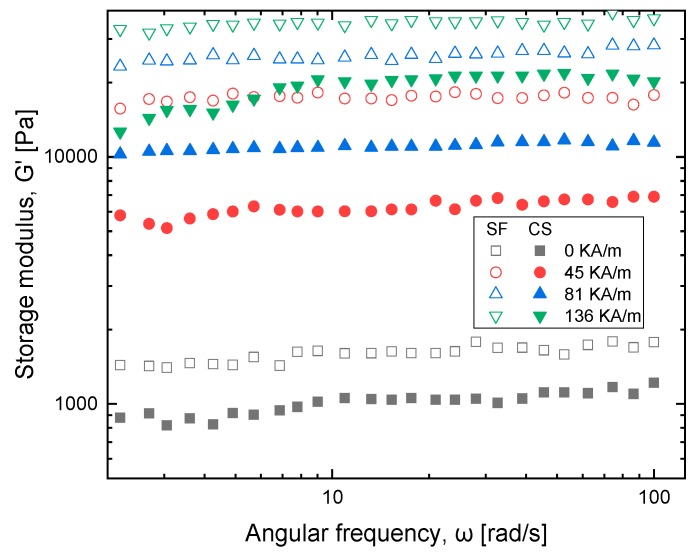
Storage modulus as a function of angular frequency at variable magnetic field strength for sunflower (SF) and cottonseed (CS) blend based MR fluid.

The viscoelastic characteristics of MR fluid under magnetic field are studied using an oscillatory test on a magneto-rheometer under different magnetic fields: 0, 45, 81, 136 kA/m. The loss modulus amplitude measurement plot is shown in Figure 7 which describes strain in MR fluid samples due to the change in loss modulus (G″) and the change in storage modulus (G′). In Figure 8, viscoelastic properties of MR fluid samples are studied. Dynamic viscoelastic properties are investigated for strain value ranging from 0.001–100% and with the frequency range of 1 to 100 rad/s, as shown in Figure 9. The performance of cottonseed oil is better than sunflower oil with the strain values that implies the dominant semi-solid character. The behavior of the solid structure is illustrated by exposing samples to magnetic fields with higher G′ values comparing to G″ when strain value is added.

The G′ value increases with an increase in magnetic strength. In addition, the loss modulus data develops two zones, first zone loss modulus with increase up to the peak implying the development of a micro-size crack on particles which occurs in semi solid-state (G′ > G″). Once the plot reaches the second zone implying growth of bigger cracks, at the end it yields to fluidic state (G′ < G″). The plots show that both sunflower and cottonseed oil blend-based MR fluid with 0.5 weight % of oleic acid respond similarly to the externally applied magnetic field with an increase in shear stress corresponding to the magnetic field, which is also observed in MR fluid characterization [20,21]. The commercially available MR fluid shows similar behavior for viscoelastic characterization which implies the fluid can be considered as a substitute considering the cost factor.

### 3.4. Sedimentation Property

**Figure 10:**
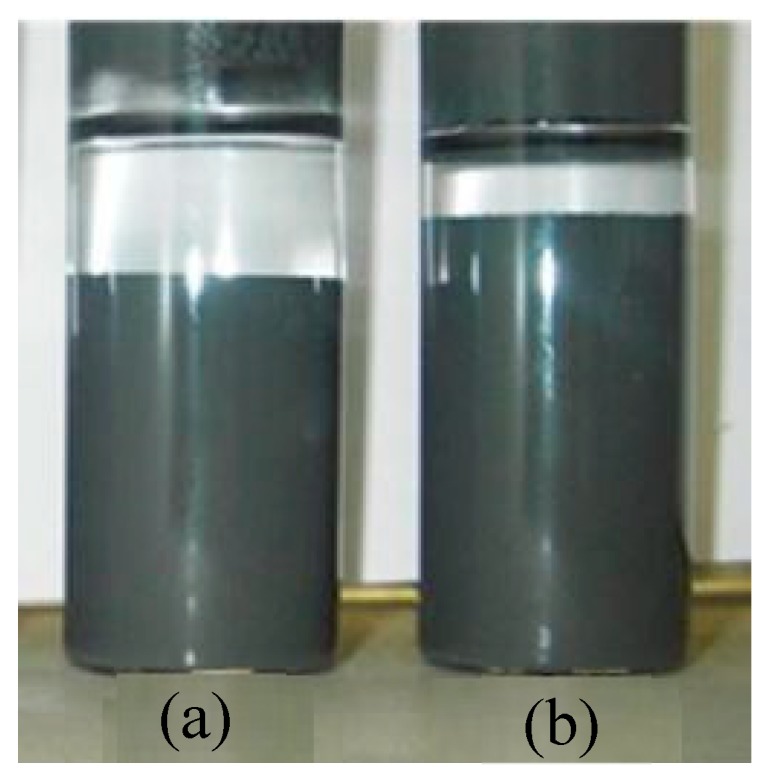
MR fluid samples (**a**) Sunflower oil blend-based (**b**) cottonseed oil blend-based.

**Figure 11:**
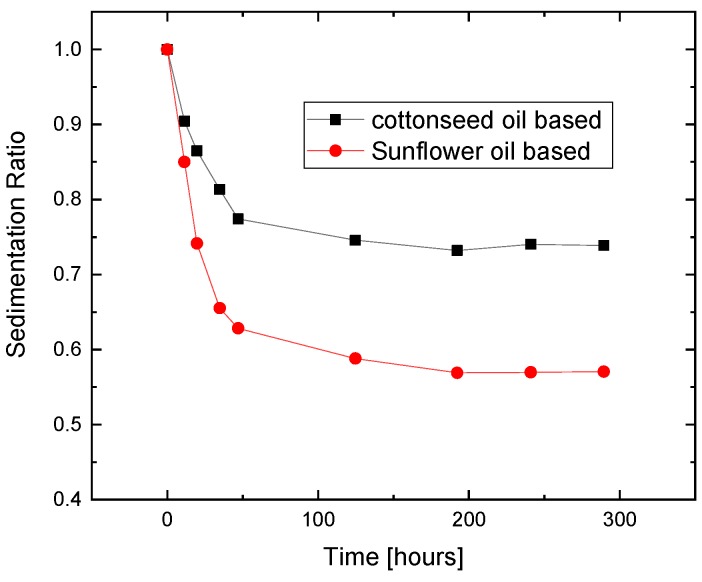
Sedimentation ratio as a function of time for the organic oil blend-based MR fluid samples.

The investigation on the dispersibility of particles in carrier fluid for both the samples is done using Turbiscan stability index. The particle suspension samples are transferred into a cylindrical cell as shown in Figure 10 and are analyzed using a light beam. The wavelength λ = 880 nm of infrared light from the source which scans sample cell which is 27.5 mm in external diameter and 70 mm in height intermittently from bottom to top. Two of the synchronous optical sensors, backscattering (BS) and transmission (T), receive the light passing across the sample which is scattered backward, respectively. The plot of the graph for the sedimentation ratio ranging from 0 to 1 along y-axis versus time along x-axis, is shown in Figure 11. The transmission data of light source for a fixed height of cell is picked to indicate the sedimentation ratio recorded as a function of time. It is observed that the sedimentation of particles occurs due to higher particle density compared to carrier fluid. As the sedimentation takes place, the transmission profile varies concerning the height of the cell and elapsed time. The sedimentation of particles in samples is shown in Figure 11 for sunflower and cottonseed oil blend-based MR fluid samples. It can be seen that in sunflower blend-based MR fluid the rate of sedimentation is faster than cottonseed blend-based MR fluid.

The guar gum coating over particles and OA facilitates suspension with the variation in viscosity of blends which are used as carrier fluid. This is considered as one of the influencing factors among the samples. Sedimentation in the MR fluid needs to be controlled to the lowest possible value, as the rate of sedimentation has a direct effect on the reduction in yield strength value. The sedimentation of Lord MRF-132DG shows a sedimentation ratio of 2.3 for a period of 72 h [22], while MR fluids synthesized in this work exhibit the sedimentation ratio between 0 to 1. The higher the sedimentation ratio, the better is the stability of magnetic particles in MR fluid. It is very clear that the performance of the synthesized samples is far better in terms of sedimentation. Thus, from the sedimentation point of view, both sunflower and cottonseed oil blend-based MR fluid performs superior to MRF-132DG.

## 4. Conclusions

This work focused on the carrier fluid in the manufacturing of new MR fluid. The carrier fluid was blended with three different fluids; silicon oil (25%), honey (25%) and organic oil (50%). Two types of organic oil are employed for the comparison purpose; sunflower oil and cottonseed oil. The reason for the choice of such natural organic oils is closely related to the eco-friendly environment which has not been considered in existing MR fluids, including commercially available MR fluid. The MR fluid characterization was studied for various compositions of oleic acid to optimize the value which was identified by 0.25% and hence this was used to synthesize the final two samples for the rheological and sedimentation study. The results obtained in this experimental work demonstrate that the prepared samples show solid-like behavior under an external magnetic field. In addition, the coating of guar gum to the particles yields better sedimentation stability. It was also identified that the cottonseed oil-based MR fluid shows better sedimentation resistance compared to sunflower-based MR fluid. It is finally remarked that MR fluids proposed in this work are eco-friendly due to the use of edible organic oils. More specifically, considering the environmental aspect, the synthesized MR fluid consists of more than 75% organic ingredients which do not cause any harm to the environment. This environmental issue has not been reported yet in the development of MR fluids. In addition, it should be remarked that the proposed MR fluids can provide comparable field-dependent rheological properties such as the storage modulus with cost-effectiveness. In the future, an optimal recipe to make eco-friendly MR fluids will be further explored with certain applications such as MR brake and MR damper.

It is finally remarked that the field-dependent rheological properties of the synthesized MR fluids need to be identified at a wide temperature range and a comparative work on various properties (such as the yield stress and wear) between commercial MR fluids and proposed MR fluids should be undertaken to demonstrate advantages and disadvantages of the proposed MR fluids. These studies will be performed as a second phase of this work.

## Figures and Tables

**Table 1:** Physical properties of components of carrier fluid blends.

	Viscosity (Pa.s)	Density (kg/m^3^)	Flash Point (°C)	Fire Point (°C)
Silicon oil	0.340	965	300	350
Cotton seed oil	0.353	926	315	371
Sunflower oil	0.0353	924	315	371
Honey	9.3	1386	40	55

**Table 2:** Physical properties of blends prepared for the carrier fluid.

	Viscosity (Pa.s)	Density (kg/m^3^)	Flash Point (°C)	Fire Point (°C)
Cotton seed (CS) oil-based blend	0.578	987	230	294
Sunflower (SF) oil-based blend	0.463	954	227	289
